# Maternal serum and cord blood leptin concentrations at delivery

**DOI:** 10.1371/journal.pone.0224863

**Published:** 2019-11-07

**Authors:** Małgorzata Stefaniak, Ewa Dmoch-Gajzlerska, Barbara Mazurkiewicz, Wanda Gajzlerska-Majewska

**Affiliations:** 1 Department of Obstetrics and Gynecology Didactics, Faculty of Health Sciences, Medical University of Warsaw, Warsaw, Poland; 2 Department of Obstetrics and Gynecology, First Faculty of Medicine, Medical University of Warsaw, Warsaw, Poland; Ospedale dei Bambini Vittore Buzzi, ITALY

## Abstract

**Introduction:**

Studies have demonstrated leptin involvement in the physiology and pathophysiology of pregnancy and suggest that leptin may be a prognostic marker for some complications of pregnancy although the association remains unclear. To date no studies have reported leptin reference intervals established in normal pregnancy, which could be used for interpreting the differences in leptin levels found in normal and pathological pregnancies.

**Objective:**

To determine leptin concentrations at delivery, in maternal serum in normal pregnancy and in cord blood and to establish reference intervals for leptin.

**Material and methods:**

The study was performed in 194 pregnant women without any comorbid health conditions. Leptin concentrations in maternal serum and in cord blood were measured by ELISA and subsequently analyzed by gestational age (weeks), maternal Body Mass Index (BMI), mode of delivery and infant gender and birth weight. For comparative analyses of normally distributed variables, parametric tests such as the Student–t were used to test the assumption of homogeneity or non-homogeneity of variance and a One-Way ANOVA when more than two groups were compared. The Pearson correlation coefficient was calculated to assess the correlation between normally distributed variables (p<0.05). The reference intervals for leptin were obtained by referring to the central 95% of laboratory test values.

**Results:**

In normal pregnant women, the mean serum leptin concentration at delivery was 37.17 ± 28.07 ng/mL and the established reference interval was 33.19–41.14 ng/mL. The mean leptin concentration in cord blood was 14.78 ± 15.97 ng/mL and the established reference interval was 12.32–17.67 ng/mL. There was a statistically significant positive correlation between maternal serum and cord blood leptin concentrations (r = 0.37; p = 0.00). Mean leptin concentrations in cord blood increased with gestational age (p = 0.00). No statistically significant differences in maternal serum and cord blood leptin concentrations were found in regard to mode of delivery and neonatal gender. A statistically significant correlation was found between maternal serum leptin and third-trimester BMI (r = 0.22; p = 0.00), but there was no association between maternal BMI and cord blood leptin concentration. There was a statistically significant positive correlation between cord blood leptin concentration and birth weight (r = 0.23; p = 0.00).

**Conclusions:**

Reference intervals for leptin in maternal serum and in cord blood established in normal pregnancy could be used in clinical practice for interpreting the differences in leptin concentrations found in normal pregnancy and in complications of pregnancy. The results indicate a strong association between maternal serum leptin levels and obesity and between cord blood leptin levels and birth weight.

## Introduction

Global obesity has become a major public health problem [1] and as a consequence the number of obese women who become pregnant is rising every year [2]. Maternal obesity is associated with a number of factors which control lipid and carbohydrate metabolism and can affect the course of pregnancy. Of these factors, leptin plays an important role in energy metabolism and fetal development [3]. Leptin is of key importance during the first stages of pregnancy since it modulates such processes as proliferation, protein synthesis, invasion and apoptosis in placental cells which are critical for normal development of the placenta [4]. The deregulated production of leptin is associated with disorders of carbohydrate metabolism with the resulting accumulation of adipose tissue and overweight or obesity. Maternal obesity carries increased risks of pregnancy complications including gestational diabetes and preeclampsia [5, 6], macrosomia, fetal growth restriction, intrauterine fetal death and stillbirth [7, 8]. Also, there is evidence linking maternal obesity to an increased risk for the offspring of developing obesity later in life [9]. Serum leptin concentrations rise during pregnancy due to weight gain and leptin expression in placental and fetal tissues [10]. Leptin concentrations peak at approximately 28 weeks and are subsequently maintained at a fairly stable level to decrease dramatically to pre-pregnancy values within the first 24 hours postpartum [11,12], which suggests a functional role of leptin during pregnancy. For decades changes in serum leptin concentrations throughout pregnancy have been of interest to researchers and clinicians. Current studies confirm that normal leptin production is a factor responsible for a normal pregnancy and embryonic/fetal development. According to some authors, leptin is involved in the regulation of prenatal hematopoiesis and brain development [10]. High leptin concentrations measured in cord blood and in infant capillary blood at birth were found to correlate with birth weight [13]. Cord blood leptin concentrations are lower than maternal serum leptin concentrations which is attributed to the role of the placenta in leptin production [10,14]. To date, it is not known whether maternal serum leptin concentrations could be used as a predictor of pregnancy complications. Although there have been numerous studies reporting changes in maternal serum leptin concentrations throughout pregnancy, normal serum leptin values and factors associated with their changes across normal, healthy pregnancy remain a matter of dispute [15]. To fill this gap in knowledge, we wanted to determine the reference values for serum leptin in normal pregnancy by measuring the concentrations of leptin in the serum samples from normal pregnant women at delivery and from cord blood of their infants. Measurements at that time-point were chosen because they reflected leptin concentrations in the third trimester and also allowed concurrent blood sampling/ assessment of leptin concentrations in mother-infant pairs. The reference intervals thus determined could be used in clinical practice to interpret correctly the differences in maternal serum and cord blood leptin concentrations as indicative of either normal pregnancy or its complications [4,10,16]. This and identification of the factors responsible for changes in maternal serum and cord blood concentrations of leptin could aid in the prevention and early treatment of adulthood metabolic and cardiovascular diseases related to altered fetal development [10].

## Material and methods

A total of 194 women in the first stage of labor were included in the study. They were identified as having a normal pregnancy and admitted for delivery to two hospitals in Warsaw, Poland, St. Sophia Hospital and the Transfiguration Hospital, in the period between January 2015 and June 2017. Participants consented in writing to the use of their samples for research.

The inclusion criteria were: age ≥18 years; first childbirth; 37 completed weeks of gestation to 40 weeks of a singleton pregnancy identified as normal; admission in the first stage of labor; subject’s consent for blood collection at delivery (samples of maternal venous blood and neonate’s cord blood) for subsequent measurement of leptin levels. Gestational age was calculated from the first day of the last menstrual period. Women with normal pregnancies only were included in the study and their pre-pregnancy Body Mass Index (BMI) was in the 18.5 to 24.9 range. Their infants were neither small nor large for gestational age. Women who did not meet the above criteria, in particular those not in active labor, pregnant with multiples or with comorbidities complicating their pregnancy were not included in the study. The current (BMI) was calculated using maternal weight and height recorded on admission.

Maternal blood samples (9 mL) were collected from an antecubital vein on admission. All women were in the first stage of labor, although not in the same phase. Fetal blood samples (9 mL) were collected from the umbilical vein immediately after delivery. Blood samples were collected into tubes containing a clot activator and serum gel separator. After centrifugation of full blood, sera were stored at -80°C until leptin measurement. Measurements were performed by the immunoenzymatic test ELISA using commercial kits (R&R System Bio-Techne) according to the manufacturer’s instructions, at the Department of General and Experimental Pathology, Medical University of Warsaw. The assay employed a monoclonal antibody specific for human leptin coated on a 96-well microplate.

1. Immediately before the assay, each serum sample was diluted 1:100 with 1 x Calibrator Diluent RDSP (10 μL of serum sample and 999 μL of 1 x Calibrator Diluent).
2. 100 μL of the Assay Diluent RD1-19 was added to each well.
3. 100 μL of serum sample and 100 μg of Standard provided by the manufacturer were added to each well and the plate was incubated for 2 hours at room temperature.
4. After incubation, the supernatant was aspirated from wells which were then washed 4 times with 400 μL of Wash Buffer.
5. After the last wash, 200 μL of Leptin Conjugate (antileptin antibodies conjugated with the horseradish peroxidase) was added to each well and the plate was incubated for 1 hour at room temperature.
6. After incubation, the supernatant was aspirated from wells which were then washed four times.
7. 200 μL of Substrate Solution (substrate for horseradish peroxidase) was added to each well and the plate was incubated for 30 minutes at room temperature, in the dark.
8. After incubation, 50 μL of Stop Solution (sulfuric acid) was added to each well.
9. The absorbance of the enzyme reaction product of each well was read at 450 nm using the ASYS UM340 microplate reader.
10. Based on the absorbance of each well with standard solutions, the standard curve was generated and subsequently used to calculate leptin concentrations in serum samples.

For a sample size of 25 μL, the detection limit was 0.17 ± 2 SD. All samples were run in duplicate and the coefficient of variation (CV) cut-off point for each duplicate was 15%.

Statistical analysis was performed with STATISTICA PL package (StatSoft, Poland). Descriptive statistics were used to present descriptions of quantitative variables. For comparative analyses of normally distributed variables, parametric tests such as the Student- t- were used to test the assumption of homogeneity or non-homogeneity of variance and a One-Way ANOVA when more than two groups were compared. The Pearson correlation coefficient was calculated to assess the correlation between normally distributed variables (p<0.05).

The reference intervals for leptin were obtained according to the Clinical and Laboratory Standards Institute (CLSI) guidelines by referring to the central 95% of laboratory test values [17].

The study was approved by the Bioethics Committee at the Medical University of Warsaw.

## Results

The mean age of study subjects was 30.0 ± 5.2 years (range: 18–44); the mean third-trimester BMI was 28.2 ± 4.1 and the mean gestational age at delivery 38.9 ± 1.1weeks. The mean birth weight was 3 354 ± 500.9 grams (g) and birth length 54 ± 2.7 centimeters (cm) (Table 1).

**Table 1:** Study group characteristics.

	Mean	Min	Max	25th quartile	75th quartile	SD
Maternal age (years)	30.0	18.0	44.0	26.0	34.0	5.2
Third-trimester BMI (kg/m^2^)*	28.1	17.5	40.2	25.2	30.7	4.1
Gestational age at delivery (weeks)	38.9	37.0	40.0	38.0	40.0	1.1
Birth weight (g)	3 454.3	1 480.0	5 030.0	3 190.0	3 780.0	503.9
Birth length (cm)	54.9	44.0	62.0	54.0	57.0	2.7

*kg/m^2^ –kilograms/meters^2^

The mean maternal serum leptin concentration was 37.17 ± 28.07 ng/mL (range 0.25 ng/mL– 121.2 ng/mL) and the established reference interval was 33.19 ng/mL– 41.14 ng/mL. The mean cord blood leptin concentration was 14.78 ± 15.97 ng/mL (range 0.37 ng/mL– 97.8 ng/mL) and the established reference interval was 12.63 ng/mL– 17.67 ng/mL (Table 2).

**Table 2:** Reference values for leptin concentrations in maternal serum and in cord blood (t = 11.38, p = 0.00).

Leptin concentration	N	Mean	CI -95%	CI 95%	Min	Max	Lower quartile	Upper quartile	SD
Maternal serum (ng/mL)	194	37.17	33.19	41.14	0.25	121.2	14.80	55.27	28.07
Cord blood (ng/mL)	194	14.78	12.52	17.04	0.37	97.8	4.98	18.35	15.97

The mean concentration of leptin was by 60% lower in cord blood than in maternal blood, the difference being statistically significant at p = 0.00.

**Fig 1:**
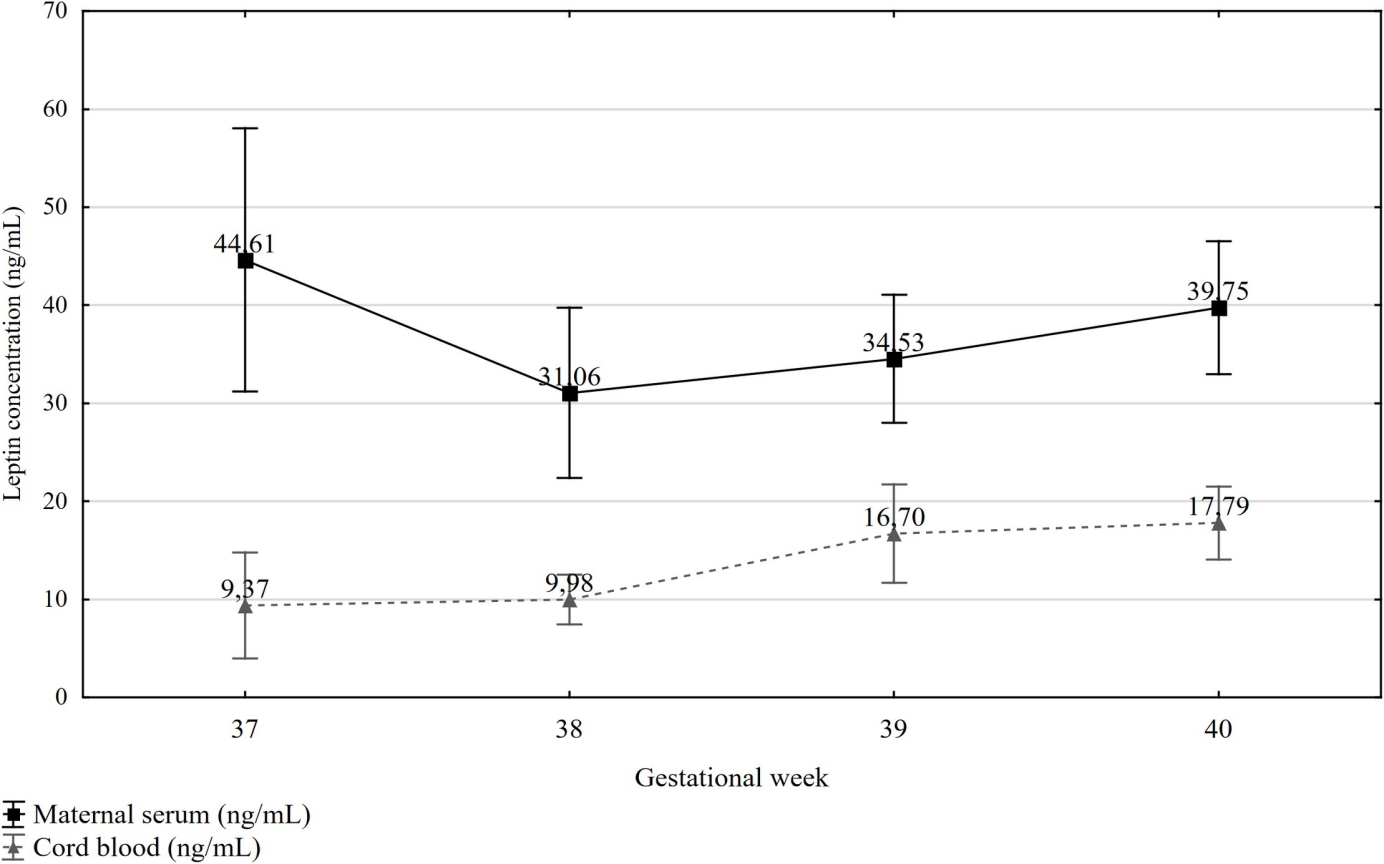
Cord blood leptin concentrations by gestational age (weeks).

The lowest mean maternal serum leptin concentration at delivery was measured at 38 weeks gestation and the highest at 37 weeks. Mean cord blood concentrations tended to increase with gestational age at delivery. At 40 weeks, the mean concentration was by 8.42 ng/mL higher than that measured at 37 weeks (Fig 1) and the difference was statistically significant (Student’s t: -1.99; p<0.02).

No statistically significant differences in maternal serum and cord blood leptin concentrations associated with neonatal gender or mode of delivery were found (Table 3). Leptin concentrations in maternal serum were strongly associated with third-trimester BMI, especially BMI>29. Maternal BMI, however, had no effect on cord blood leptin.

**Table 3:** Maternal serum leptin and cord blood leptin by neonatal gender, mode of delivery, gestational age at delivery and third-trimester BMI.

	N	Maternal serum leptin (ng/mL)			Cord blood leptin (ng/mL)		
		Mean	SD	p value	Mean	SD	p value*
**Neonatal gender**							
Female	94	34.28	25.31		13.38	10.82	
Male	100	39.89	30.31	0.16	16.10	19.58	0.24
**Mode of delivery**							
Vaginal, non-operative	129	37.83	28.04		15.48	15.29	
Emergency cesarean section	65	35.86	28.30	0.65	13.38	17.26	0.39
**Gestational age at delivery (weeks)**							
37	28	44.61	34.64		9.37	19.01	
38	36	31.06	25.63		9.98	7.49	
39	62	34.53	25.76		17.40	22.57	
40	68	39.75	27.96	0.19	17.79	15.39	0.02
**Third-trimester BMI** (kg/m^2^)							
<25	43	32.41	30.19		13.70	16.22	
<25–29>	77	32.74	25.54		15.31	17.45	
>29	74	44.54	28.12	0.02	14.86	14.30	0.87

T-test or One-Way ANOVA (F) test

A statistically significant positive correlation was found between leptin concentrations in maternal serum and in cord blood (Pearson’s r = 0.37; p = 0.00). The association was stronger for male infants (Pearson’s r = 0.44; p = 0.02) than for female infants (Pearson’s r = 0.24; p = 0.02).

**Fig 2:**
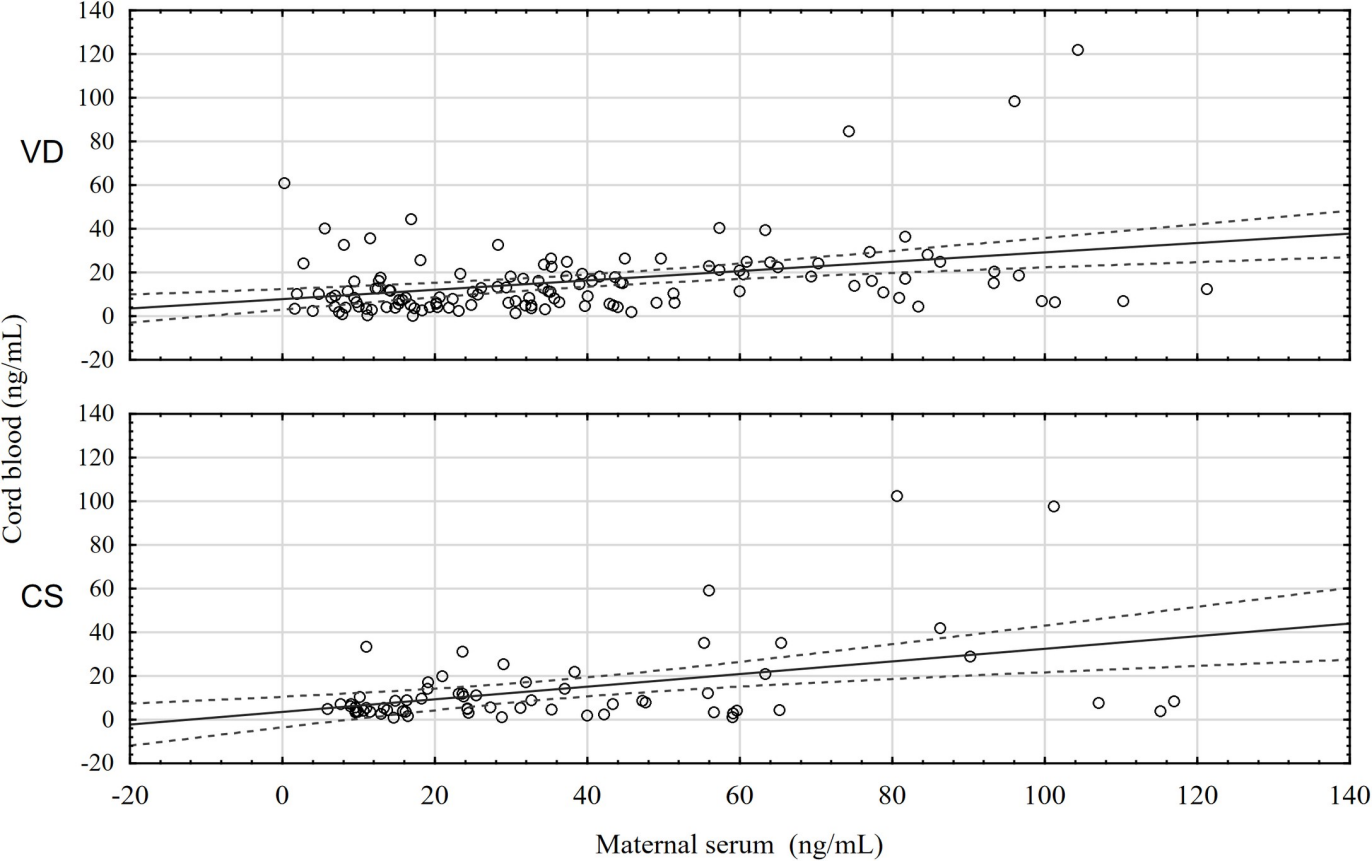
Correlation between maternal serum leptin concentration and cord blood leptin concentration by mode of delivery (VD: Pearson’s r = 0.35; p = 0.00; CS: Pearson’s r = 0.43; p = 0.00).

The correlation between maternal serum and cord blood leptin concentrations was weaker for women who had a vaginal delivery (VD) (Pearson’s r = 0.35; p = 0.00) than for those who were delivered by a cesarean section (CS) (Pearson’s r = 0.43; p = 0.00), but in both instances the correlation was statistically significant (Fig 2).

Leptin concentrations were assessed with respect to birth weight and no statistically significant correlation was established between maternal serum leptin concentration and infant birth weight. However, there was a statistically significant positive correlation between cord blood leptin concentration and infant birth weight (Pearson’s r = 0.23; p = 0.00). The correlation was significant for non-operative vaginal deliveries (Pearson’s r = 0.21; p = 0.02), but not for deliveries by a cesarean section (Pearson’s r = 0.22; p = 0.08). The correlation between cord blood leptin concentration and birth weight was stronger for female infants (Pearson’s r = 0.25; p = 0.02 than for male infants (Pearson’s r = 0.21; p = 0.04), but both correlations were statistically significant.

A statistically significant correlation was found between maternal serum leptin concentration and third- trimester BMI (Pearson’s r = 0.22; p = 0.00) but not between cord blood leptin concentration and third-trimester BMI.

## Discussion

We determined mean maternal blood leptin concentrations in the first stage of labor in a normal pregnancy and in cord blood of their infants. The highest maternal leptin level was found at 37 weeks gestation and the lowest at 38 weeks. There are very few published studies on maternal and fetal leptin concentrations determined concurrently at one time-point, that is at delivery. To the best of our knowledge, this study is one of the first evaluations involving a large and ethnically homogenous study group (194 mother-infant pairs). The literature related to this question is scarce and comparison with two earlier studies we have identified is difficult due to either a much smaller study group or subject ethnicity. In 1998, Schubring et al. assessed a small group of pregnant women and reported similar maternal serum leptin levels which peaked at 38–40 weeks of pregnancy and exceeded the cord blood leptin values [18]. Another study, by Ishrat et al [19]. found at delivery maternal serum leptin levels of 24.50 ng/mL (range 13.15–45.60) and cord blood leptin levels of 6.50 ng/mL (range 2.02–1.30) while in the present study leptin concentrations in both maternal serum and in cord blood were higher). The difference could result from the effect of ethnicity (Asian versus European subjects) with potential dissimilarities due to BMI, diet and dietary supplements.

Published studies have attributed elevated leptin levels during pregnancy to substantial increases in the amount of adipose tissue [3, 4, 20]. The results of our study confirm a correlation between maternal blood leptin and BMI. Other authors believe that increased leptin concentrations in maternal blood are due to the placental production of leptin as documented by assessment of maternal leptin levels during pregnancy and postpartum. Maternal serum leptin concentrations begin to rise in the first trimester of pregnancy, long before the weight gain associated with pregnancy becomes noticeable, and fall to the pre-pregnancy values within the first 24 hours postpartum, which suggests that the hormone is associated with maternal metabolic adaptation to pregnancy [4]. With the two sources of leptin, i.e. maternal adipose tissue and the placenta, leptin concentrations were higher in maternal blood than in cord blood as confirmed by Weyermann et al. [21]. Laml et al. also observed the difference but in their study it was not significant. They hypothesized that leptin, a 16-kDa protein, cannot cross the placenta [22]. In our study, the mean cord blood leptin concentration was nearly twice as low as the mean concentration in maternal serum. We demonstrated a statistically significant positive correlation between the mean maternal serum leptin concentration at delivery, irrespective of gestation length, and the mean cord blood leptin.

Marino-Ortega et al. observed a similar correlation [23]. We did not find any differences in leptin levels, both maternal and neonatal, associated with mode of delivery. The available literature data are not consistent. Yoshimitsu et al. found statistically higher cord blood leptin concentrations in neonates born by a vaginal delivery [24]. The differences were accounted for by augmented synthesis and release of leptin due to decreased oxygenation of blood during vaginal delivery with activation of the sympathetic system and the resulting increase in cortisol synthesis which stimulates leptin release. The above results were not confirmed by other authors. Logan et al. observed that cord blood leptin concentrations were associated with duration of labor rather than delivery mode [25]. A number of studies have shown that leptin levels are generally higher in women who have more subcutaneous adipose tissue than men while estrogen stimulates leptin release by adipocytes and acts on the hypothalamus increasing its leptin sensitivity [3, 23]. We did not observe any significant differences in leptin concentrations, either in maternal serum or in cord blood, associated with infant gender. When infant birth weights and other anthropometric measurements were similar, there were no apparent differences in leptin concentrations between genders. Some authors have observed higher cord blood leptin concentrations in female neonates and although the cause has not been convincingly explained, estrogen has been implicated as possibly increasing leptin levels in female infants [26]. This inconsistency may result from studies based a single leptin measurement with no measurements of other hormones involved in the regulation of leptin levels. We also assessed leptin levels in relation to infant birth weight and found no statistically significant association with maternal serum leptin which confirms the results obtained by other authors [19,27–29]. However, there was a statistically significant correlation between cord blood leptin and infant birth weight. This finding is confirmed by other authors who underlie the role of leptin in the regulation of appetite and metabolism, which may also lead to changes in body weight in the first days after birth and suggest that leptin could be a marker of nutritional status [25, 30–35]. A significant correlation was found between maternal BMI and maternal serum leptin concentration, but not cord blood leptin. Marino-Ortega et al. also observed a strong association of maternal leptin blood levels with weight gain during pregnancy [23]. During pregnancy, however, BMI is not a reliable indicator of increases in the amount of body fat. Shroff et al. showed a moderate correlation only between maternal leptin levels and BMI [36]. These inconsistent observations in pregnant women may suggest that the association between maternal BMI and leptin levels is not obvious and other factors or complications of pregnancy may affect leptin levels [3, 15, 37]. Further studies are needed to elucidate leptin involvement in some complications of pregnancy and to evaluate potential uses of leptin measurements as a predictor of pregnancy pathologies. Leptin may be a valuable additional marker (diagnostic tool) to use for predicting complications of pregnancy.

First, reference intervals should be obtained for leptin levels in each trimester of a normal pregnancy, followed by establishing week-by-week reference intervals in pregnancies identified as pathological. In the present study, the reference intervals for leptin were obtained by referring to the central 95% of leptin values in serum specimens from 194 healthy women at delivery and their infants. According to the Clinical and Laboratory Standards Institute (CLSI) guidelines, the sample size of 120 is the minimum number to establish a reference interval [17]. The results obtained are therefore valid and we hope that they can be used as a reference in further studies. Comparison of these sets of leptin values would be helpful in correct assessment of leptin measurements in pregnancy and could potentially lead to better understanding of the role of leptin in the pathomechanism(s) of some disorders of pregnancy or even to the use of serum leptin as a biomarker for such disorders.

### Strengths and limitations

This study is one of the few published evaluations of leptin levels assessed concurrently in maternal serum and infant cord blood, which estimated the reference values in normal pregnancy. The study group was large enough and homogenous. The obtained reference intervals for leptin in normal pregnancy when used in further studies of a variety of complications of pregnancy including intrahepatic cholestasis of pregnancy, pregnancy-induced hypertension, intrauterine growth restriction or gestational diabetes, could be assessed for use as a useful prognostic marker for any of these complications. Standardized reliable procedures were used for all laboratory and clinical measurements, including anthropometry.

The main limitation of our study is the observational study design which does not allow any conclusions describing causality or possible mechanistic pathways. All women included in the study had a BMI in the range recognized as normal and we did not analyze the findings for correlations between the pre-pregnancy BMI and leptin concentrations at delivery,. In future studies an association between pre-pregnancy BMI and leptin concentrations during pregnancy should be more thoroughly investigated.

Leptin may be a valuable additional marker (diagnostic tool) to use for predicting complications of pregnancy. However, before its clinical use could be reliably recommended, further studies are needed. It would be advisable to check the reproducibility and generalizability of the present results in cohort studies and prospective studies in larger populations of women recruited in earlier stages of pregnancy to assess maternal serum leptin for changes over time, and if possible also include the postpartum period.

Another limitation is a single maternal blood collection. As explained above, maternal blood was collected on admission to the delivery ward. All subjects were in the first stage of labor, but in different phases of that stage. Indeed, according to some authors, the time-point of collection during labor may influence leptin values. Further studies should also assess the possible effect of the time of day in which blood samples are collected as leptin is secreted into the bloodstream in a pulsatile fashion with a circadian rhythm and maximum circulating levels are reached between 1 am and 2 am and minimum levels between 8 am and 9 am. The study population was of European descent and so the obtained reference intervals might not apply to other ethnicities.

## Conclusions

1. The study established the reference intervals for leptin concentrations in the serum of healthy pregnant women at delivery and in cord blood of their infants, according to the Clinical and Laboratory Standards Institute (CLSI) guidelines.
2. The reference intervals for leptin concentrations in maternal serum and in cord blood could be potentially useful in clinical practice to assess maternal serum leptin levels as suggestive of either normal or pathological pregnancy.
3. Mean cord blood leptin concentrations increased with gestational age at birth.
4. There was no association between maternal serum leptin or cord blood leptin concentrations and infant gender or mode of delivery.
5. There was a strong correlation between third-trimester BMI and maternal serum leptin concentration at delivery.
